# Intraspherulitic Melting-Temperature Distribution of Poly(butylene 2,6-naphthalate) Containing β′-Crystals Controlled by Secondary Crystallization

**DOI:** 10.1021/acs.macromol.5c00542

**Published:** 2025-04-22

**Authors:** Mengxue Du, Katalee Jariyavidyanont, Joachim Ulrich, Christoph Schick, René Androsch

**Affiliations:** † Interdisciplinary Center for Transfer-oriented Research in Natural Sciences, Martin Luther University Halle-Wittenberg, Hoher Weg 7a, 06120 Halle/Saale, Germany; ‡ Martin Luther University Halle-Wittenberg, Hoher Weg 7a, 06120 Halle/Saale, Germany; § Institute of Physics and Competence Centre CALOR, University of Rostock, 18051 Rostock, Germany

## Abstract

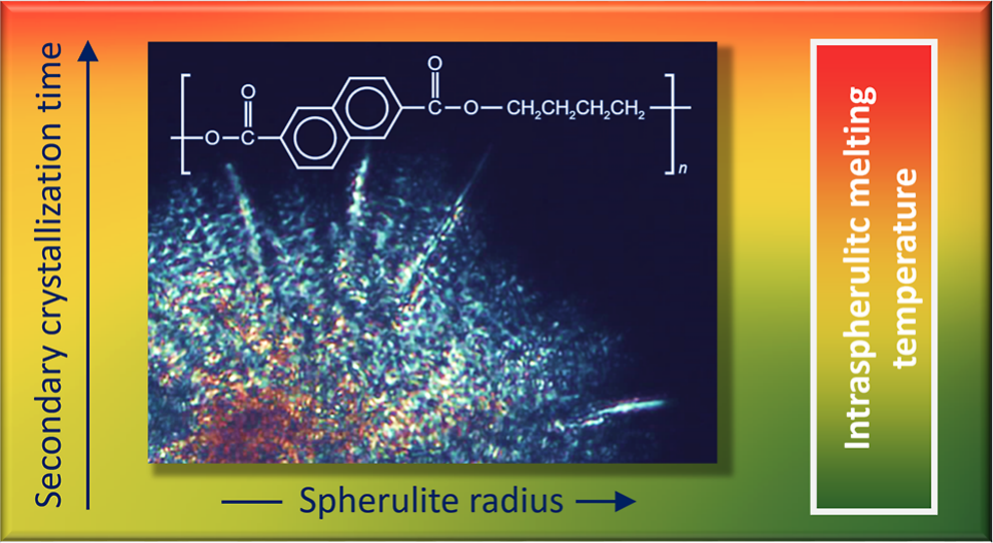

The combination of low crystal-growth rate and low nuclei density, as evident, e.g., on hot-crystallization at low melt-supercooling, allows formation of rather large spherulites containing isothermally grown crystals subjected to different times of secondary crystallization, causing an intraspherulitic melting-temperature distribution. As demonstrated on example of the β′-high-temperature-crystal polymorph of poly(butylene 2,6-naphthalate) (PBN), crystals located in the spherulite centers, subjected to annealing during the slow growth of the spherulite, melt at distinctly higher temperature than non-annealed crystals near the spherulite boundary, causing spherulite inward melting. The melting-temperature gradient along the spherulite radius, however, diminishes if all parts of the spherulites are annealed, e.g., after a space-filled spherulitic morphology is achieved, yielding a radius-independent intraspherulitic melting temperature. Otherwise, the intraspherulitic melting-temperature distribution may be preserved/frozen-in by cooling, with implications on properties due to the presence of crystals of different stabilities. Assessing the intraspherulitic melting-temperature distribution required suppression of crystal reorganization on heating, which was achieved by analysis of the heating-rate dependence of melting. These experiments confirmed the initially lower stability of crystals near the spherulite periphery by their enhanced reorganization/stabilization on sufficiently slow heating compared to crystals located in the spherulite center, being less vulnerable for reorganization. In summary, the study highlights the importance of secondary crystallization/annealing on the thermodynamic stability/melting behavior of crystals arranged in a spherulitic semicrystalline superstructure. In addition, the performed study also provides new data about the growth of radial and tangential lamellae in PBN when crystallized at low melt-supercooling.

## Introduction

Poly(butylene 2,6-naphthalate) (PBN) is a semirigid crystallizable polyester with the chain-repeat unit containing a rigid aromatic subunit and a flexible aliphatic segment. This polymer exhibits excellent hydrolysis, chemical, and thermal resistance, superior gas, water, and UV-light barrier performance, high impact strength and elongation at break,^1−4^ enabling applications in the fields of rigid packaging, thermoforms, engineering resins,^4^ and electric vehicle infrastructures, with huge prospects.^5^

PBN is polymorphic, that is, it may exist in different crystal structures depending on the crystallization conditions. In short, at rather low and high supercooling of the melt, β′-and α-crystals grow, respectively. The α-crystal growth is a multistep process involving the temporary formation of a liquid crystalline phase, which, however, can be isolated by quenching.^6−8^ PBN β′-crystals, being the focus of the present work, exhibit a triclinic unit cell,^9−13^ with the equilibrium melting temperature (*T*_m_^0^) being 281 °C.^12^ The formation of β′-crystals is controlled by temperature such that it grows on cooling the quiescent melt at rates lower than about 1 K/min or at temperatures higher than about 230 °C.^6,11−16^ In this work, we continue our study of β′-crystals^15,16^ and investigate their melting and reorganization behaviors. Beyond the academic interest in this research, it is worth noting that β′-crystals have also been detected after industrial processing of PBN by injection molding.^17^ Knowledge of the thermal stability of such crystals is therefore required to fully exploit the applicability of this high-performance polymer.

Regarding crystal stability, slow heating of nonequilibrium polymer crystals of low metastability, typically grown well below *T*_m_^0^, leads to their melting a few K above the temperature of formation. This melting may be followed by fast recrystallization and remelting, often though not always causing multiple melting events.^18−25^ Crystal-reorganization is time-dependent and may be suppressed by fast heating.^21−25^ Also in the case of PBN, multiple melting events are detected during slow heating when exposed to specific crystallization routes.^14,26,27^ Ju and Chang^14^ found two melting peaks in differential scanning calorimetry (DSC) curves recorded on heating at 10 K/min after isothermal crystallization at different temperatures, suggesting that the low-temperature melting peak is caused by crystals formed during isothermal crystallization, while the high-temperature melting event relates to recrystallized species formed during heating. Yasuniwa et al.^26,27^ confirmed—by variation of the initial structure (controlled by the crystallization temperature) and by variation of the heating rate—that the double melting of PBN is caused by recrystallization of α-crystals and proposed that there is no change in the crystal structure during melting. Though a small amount of β′-crystals was detected after high-temperature crystallization, dedicated experiments for analysis of their melting and possible reorganization were not performed.

In this study, we employed hot-stage polarized-light optical microscopy (POM) to continue the initial works on PBN crystal reorganization during slow heating,^14,26,27^ briefly described above, focusing on the behavior of currently under-investigated β′-crystals of inherently different stability and morphology than α-crystals. Besides further completion of crystal-reorganization studies of polymorphic PBN, with the employment of hot-stage POM, we also attempt to provide new insights into the understanding of polymer melting on the micrometer scale. For example, reorganization of crystals of poly(butylene terephthalate), poly(ethylene terephthalate) (PET), poly(ethylene naphthalate), or poly(ether ether ketone) on slow heating appeared independent of the radial spherulite position, as followed by light-intensity measurements or analysis of the birefringence in POM.^28−30^ Considering the presence of crystals of PET grown during primary and secondary crystallization, Medellin–Rodriguez et al.^30^ found sequential growth of dominant lamellae and subsidiary branches during isothermal crystallization, with branching presumably caused by the rejection of material from the dominant lamellae or by initially noncrystalline structure near the dominant lamellae. The melting process of such structures is then considered as the reverse of the isothermal crystallization process such that the secondarily grown branches disappear first before the melting of the dominant lamellae, however, again, independent of the position in spherulites. For poly(l-lactic acid) (PLLA), in contrast, isothermal melting/reorganization proceeded by an initial spherulite inward melting process, allowing, however, reorganization and stabilization of the central part of the spherulite by lamellar thickening, slowing down the melting rate.^31^

In the special case of presence of the polymerization-initiator Salen–Al–OCH_3_, spherulites of PLA simply “dissolve” along the radius toward the center, without reorganization.^32^ Even more complicated spherulite melting and reorganization patterns are reported for isotactic polypropylene (iPP) containing crosshatched lamellae,^33−35^ as well as for sc-PLA [blends of PLLA and poly(d-lactic acid)].^36^ Regarding iPP, the highest melting temperature was detected for dominant radial lamellae far from the center of spherulites, while evenly crosshatched spherulite centers showed a lower melting temperature due to suppression of isothermal lamellar thickening.^34^ For PBN β′-form spherulites grown on slow cooling the melt at 0.1 K/min, subsequent heating at 10 K/min first caused the disappearance of secondary-grown crystals, leaving the spherulite skeleton for observation, without detection and discussion of possible reorganization,^13^ further investigated here.

## Experimental Section

Additive-free PBN pellets with an intrinsic viscosity of 0.92 dL/g,^8^ measured at 30 °C using a 60:40 m/m % mixture of phenol and 1,1,2,2-tetrachloroethane were provided by Teijin Shoji Europe GmbH (Hamburg, Germany). Throughout all crystallization experiments, the polymer was heated to 290 °C to eliminate any prior thermal history and to obtain an equilibrated/relaxed melt.

To subject PBN to specific crystallization routes and to investigate its melting behavior on slow heating, we employed a calibrated heat-flux DSC 1 (Mettler-Toledo, Greifensee, Switzerland) equipped with an FRS 5 ceramic sensor, with the DSC connected to an intracooler TC100 (Huber, Offenbach, Germany). We used nitrogen gas at a flow rate of 60 mL/min for purging the furnace and 40 μL-aluminum pans for encapsulation samples with masses around 3–5 mg. To prove secondary crystallization at the temperature of primary isothermal crystal growth, we performed rapid heating calorimetric experiments in which crystal reorganization was suppressed and which allowed the melting temperature to be determined as a function of the crystallization time. For this, we used a Flash DSC 2+ fast scanning chip calorimeter (Mettler-Toledo, Greifensee, Switzerland) in combination with UFH-1 chip sensors. The instrument was operated in conjunction with an intracooler TC100, and the sample environment was purged with nitrogen gas at a flow rate of 35 mL/min. Sample preparation involved cutting 8 μm thin sections from the as-received pellets using a microtome and reducing their lateral dimension to around 10–20 μm using a scalpel and a stereomicroscope.

For POM imaging, we employed a DMRX microscope (Leica, Wetzlar, Germany) equipped with a Motic CCD camera and a THMS600 hot-stage (Linkam, Tadworth, UK). The microscope was operated in transmission mode, with 10 μm thick specimens prepared using a rotary microtome (Slee medical GmbH, Niederolm, Germany) and placed between crossed-polarizers.

X-ray diffraction (XRD) measurements served for gaining information about the presence of specific crystal polymorphs and were performed in transmission mode employing a Retro-F laboratory setup (SAXSLAB, Copenhagen, Denmark) in combination with a microfocus X-ray source and an ASTIX multilayer X-ray optics (AXO Dresden GmbH, Dresden, Germany) as monochromator, providing CuKα radiation with a wavelength of 0.154 nm. The size of the approximately circular X-ray beam, generated by a double-slit, was around 0.9 mm, and the intensity of the scattered X-rays was measured by a two-dimensional (2D) PILATUS3 R 300 K detector (DECTRIS Ltd., Baden, Switzerland), with the 2D X-ray patterns azimuthally averaged to obtain the intensity as a function of the scattering angle 2θ. Preparation of samples for X-ray analysis included compression-molding of films of about 200 μm thickness with a film-maker accessory (Specac Ltd., Orpington, UK) in combination with a heatable hydraulic press (LOT QD, Darmstadt, Germany). Afterward, a specimen was inserted into a 20 μL-aluminum pan (Mettler-Toledo, Greifensee, Switzerland), which, finally, was attached to the silver-block of an HFS350 hot-stage (Linkam, Tadworth, UK), serving as a sample holder in the X-ray setup. For monitoring of the sample temperature, a μ-thermocouple (Omega Engineering GmbH, Deckenpfronn, Germany) was attached to the sample pan and connected to a fast OM-DAQXL-1-EU data logger (Omega Engineering GmbH, Deckenpfronn, Germany).

## Results and Discussion

### Isothermal Crystallization of PBN to Obtain β′-Crystals

**Figure 1:**
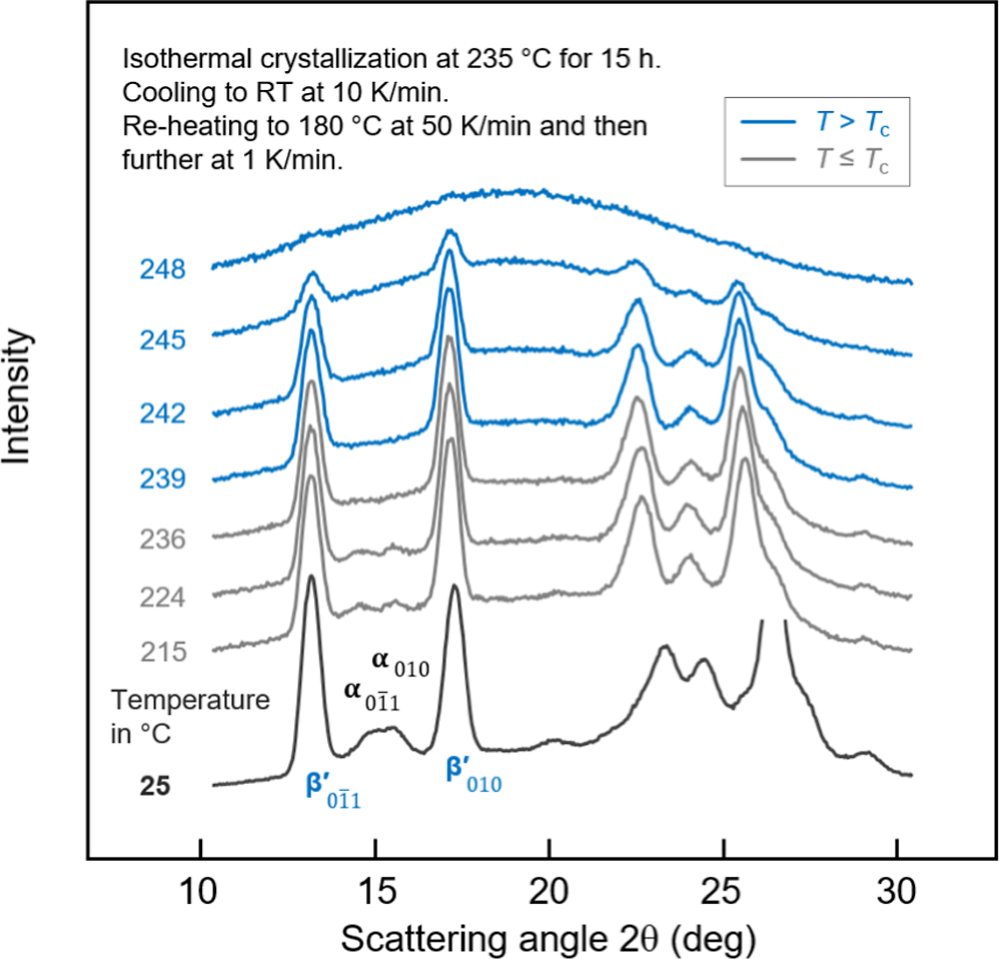
XRD curves, intensity as a function of the scattering angle 2θ, of PBN crystallized at 235 °C and containing β′-crystals, collected on heating at 1 K/min. Selected β′- and α-peaks are indicated. Gray and blue coloring of curves indicates measurement-temperatures *T* below and above the crystallization temperature *T*_c_ of 235 °C, respectively.

To understand the melting/reorganization behaviors of PBN predominantly containing β′-crystals, in a first experiment, XRD measurements were performed to monitor structural changes of an isothermally crystallized sample during slow heating. Figure 1 shows a set of XRD curves of a sample isothermally crystallized at 235 °C for 15 h, followed by cooling at 10 K/min to room temperature (RT). The bottom dark-gray curve represents data collected at RT, while the light-gray and blue curves refer to measurements performed during continuous heating at 1 K/min below and above the crystallization temperature *T*_c_ of 235 °C, respectively.

The XRD scan collected at 25 °C reveals the presence of a large fraction of β′-crystals, as indicated, but also the presence of a few α-crystals. These α-crystals formed during cooling to RT at a lower temperature, as the corresponding 01̅1 and 010 diffraction peaks disappear on heating before reaching the β′-crystallization temperature of 235 °C. Note that DSC analyses provided additional evidence of minor crystallization during cooling PBN after long-term high-temperature crystallization at 235 °C (not shown). The β′-crystals then start to melt around 239 °C, as detected by the lowering of the intensity of the corresponding diffraction peaks, with the melting process stretching to almost 248 °C. Note that the experiment of Figure 1 has not been performed to identify possible reorganization, impossible at the selected temperature resolution, rather than to confirm the exclusive presence of β′-crystals on crystallization at 235 °C.

**Figure 2:**
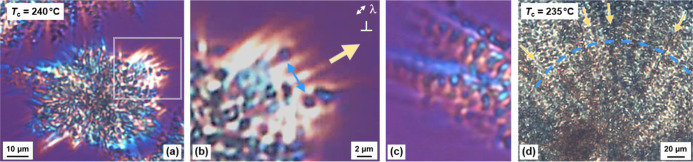
POM micrographs of PBN containing β′-crystals. (a) Incompletely at 240 °C within 7–8 h grown spherulite with the image taken at 240 °C. (b) Soft-zoom of the framed part of the spherulite in (a). (c) Detail of spherulite growth at 240 °C. (d) Spherulite grown at 235 °C for about 3.5 h, with the image taken at 235 °C. Images (a–c) were captured with an inserted λ-plate with its orientation provided in (b). Images (a,b) are adapted under the terms of the CC-BY license 4.0 (https://creativecommons.org/licenses/by/4.0/), Copyright 2024, M. Du, R. Androsch, D. Cavallo, published by Wiley (10.1002/pol.20230810).^41^

For demonstration of the peculiar growth of β′-crystals, Figure 2 shows POM micrographs of PBN with (a) an incomplete PBN spherulite grown within 7–8 h at 240 °C, (b) a soft-zoom of the framed part of the spherulite in (a), (c) a detail of spherulite growth at 240 °C, and (d) a spherulite grown at 235 °C within about 3.5 h. Note, all images were taken at 240 °C (a–c) or 235 °C (d). Furthermore, images (a–c) were captured with an inserted λ-plate with its orientation provided in image (b). Starting from a nucleus (not visible), in early stages of the crystallization process, birefringent streak-like domains grow into the melt, with their orange and blue colors, when imaged with an inserted λ-retardation plate, suggesting orientation of molecules perpendicular to the growth direction, with the latter indicated with the dark yellow arrow in image (b). Whether these domains are crystals or crystallization precursors/oriented structures, as perhaps indicated with the fading birefringence/coloring into the melt, is unknown. The sample volume between these streak-like domains is then filled up by additional crystals. The chain orientation of these additional crystals is approximately perpendicular to the chain segments in the initial needle-like structures, leading to an overall spotty appearance. The nearly perpendicular orientation of these crystals compared to the initial structures is indicated by their blue color in image (b). As such, there are evident two populations of crystals: (i) long radial lamellae growing into the melt and (ii) shorter tangential lamellae, filling the space between the initial needles. Such perpendicular growth of daughter on mother lamellae, also shown in image (c), leads to a dendritic appearance of the structure, in particular, at the growth front. However, the used terminology of mother and daughter lamellae does not necessarily imply, but also does not exclude, epitaxial growth/branching as in iPP.^37,38^ In any case, the images do not suggest low-angle branching or branching by tip-splitting.^39,40^ The image (d) finally shows the spherulite structure after space-filling, with radial and tangential lamellae not as easy to identify as after incomplete crystallization. Nevertheless, careful inspection still allows recognition of the long structures parallel to the spherulite radius (see dark-yellow arrows) and tangentially grown crystals with an apparently circular symmetry (see the blue dashed line).

### Melting of PBN β′-Crystals at Different Heating Rates

**Figure 3:**
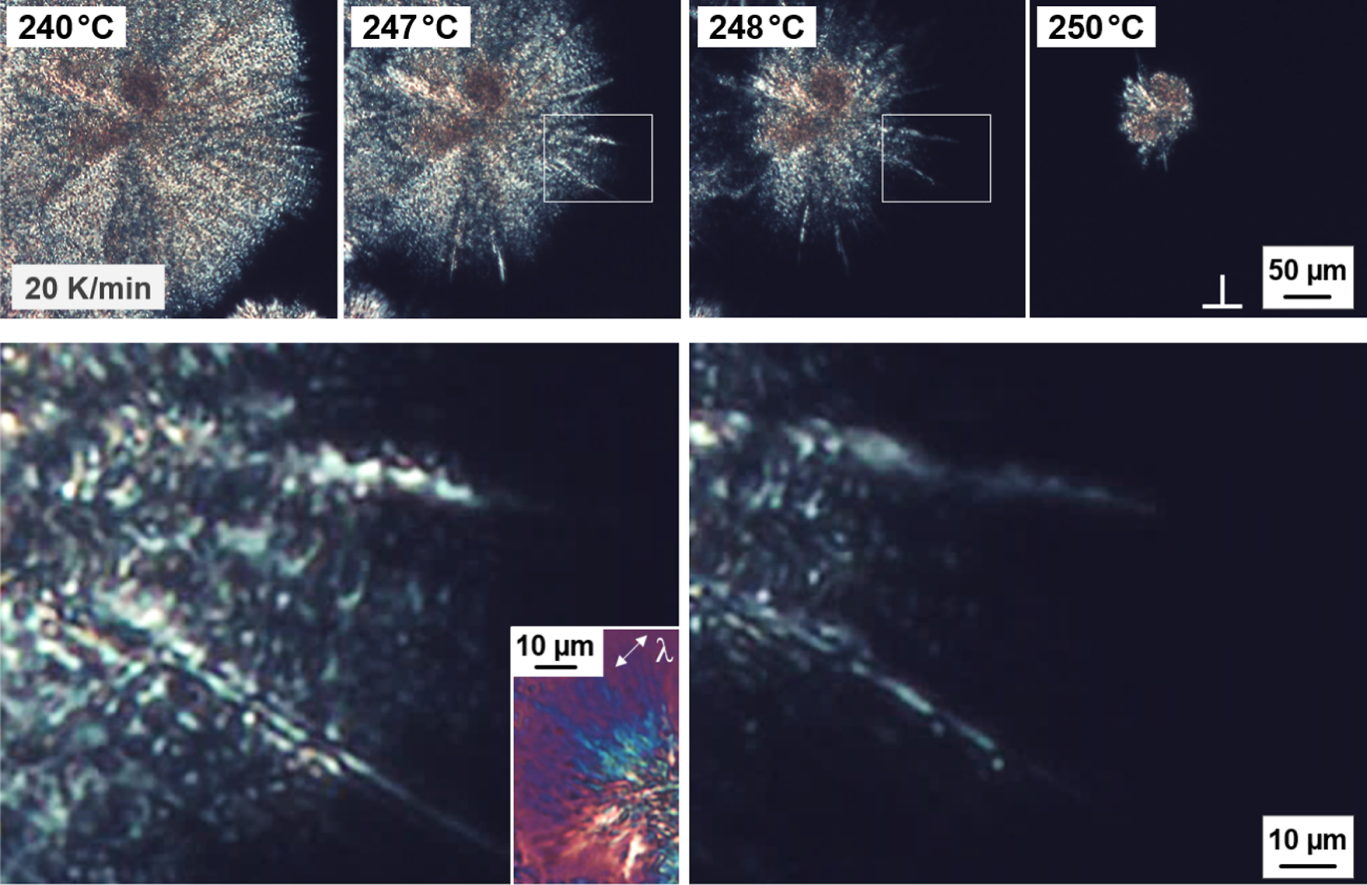
POM micrographs of PBN containing β′-crystals formed at 235 °C, obtained during heating at 20 K/min (upper row). The left and right lower-row images are soft-zooms of the framed area in the images collected at 247 and 248 °C, respectively, for illustration of melting of tangential and radial crystals. Worth noting that crystallization at 235 °C was interrupted before space-filling, that is, secondary crystallization was minimized, at least for the outer spherulite parts.

Figure 3 illustrates the melting process of a PBN spherulite, grown for 3.5 h at 235 °C and containing β′-crystals, during heating at 20 K/min, with the images to be read from left to right. The upper-row images provide an overview at a lower magnification while the two lower images show details corresponding to the framed area in the micrographs collected at 247 and 248 °C. The inset in the lower left micrograph shows a part of a spherulite, which is captured at the same temperature (247 °C) with an inserted λ-plate. The images suggest that melting starts at the spherulite boundary and proceeds toward the spherulite center, which appears to be the most stable part of the spherulite (see the upper-row image collected at 250 °C). Moreover, it appears that tangential lamellae exhibit a slightly lower stability than the radial lamellae for a given radial distance, disappearing first and then unmasking the radially grown needle-like crystals (see the two lower images), confirming earlier works reported elsewhere.^13^

**Figure 4:**
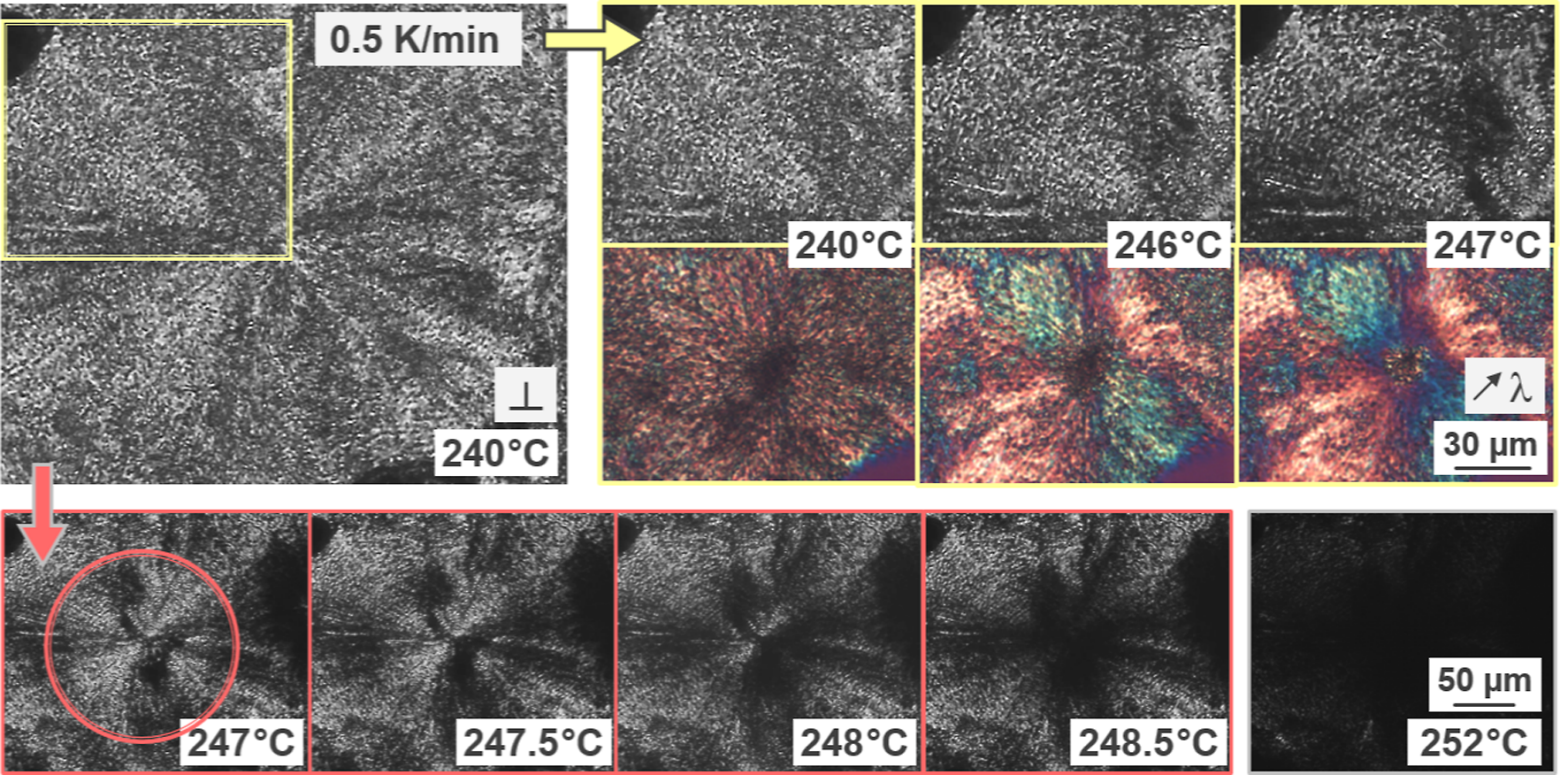
POM micrographs of PBN spherulites containing β′-crystals formed at 235 °C, collected during heating at 0.5 K/min. Yellow- and red-framed images refer to temperature-ranges from 240 to 247 °C and from 247 to 248.5 °C, respectively. Further explanations are provided in the text.

Next, spherulites containing β′-crystals, prepared at the same conditions as in Figure 3, were heated at a low rate of 0.5 K/min until complete melting. In contrast to melting during relatively fast heating at 20 K/min (see Figure 3), slow heating, in part, allows reorganization of β′-crystals, depending on the radial position within the spherulites. For illustration, the top left POM image of Figure 4 shows a large spherulite with a diameter of around 250 μm, while the yellow- and red-framed images were captured during heating of this large spherulite, covering the temperature ranges from 240 to 247 °C and from 247 to 248.5 °C, respectively. Furthermore, the images between 240 and 247 °C were collected without (top row yellow-framed grayscale images) and with an inserted λ-retardation plate (bottom row yellow-framed color images), for the sake of improved contrast. In this temperature-range, the integrity of the spherulite seems preserved, however, with the turn of colors in the various spherulite-sectors, indicating improved alignment of crystals parallel to the radius, holding, in particular, for the spherulite outer regions. Subsequent to this initial change of the spherulite morphology, with the temperature further increasing to 248.5 °C, the inner parts of the spherulites (see the red dashed circle) gradually melt, with minor local differences regarding the melting temperature. However, in the outer parts of the spherulites, grown in the late stage of the crystallization process, still considerable amount of nonmolten material is left. As such, it appears that initially low-stability crystals in regions far from the spherulite centers gain stability during slow heating, in agreement with the observed contrast-change of POM images collected with an inserted λ-plate below 247 °C. Finally, the spherulites fully melt when the temperature reaches 252 °C, as indicated with gray-framed bottom right image. Qualitatively, the reproducibility of observations was confirmed by analyses of several samples. This notwithstanding, slight differences in the observed melting temperature (of the order of magnitude up to around 1 K) are reported, which, however, do not affect the general interpretation of the data.

**Figure 5:**
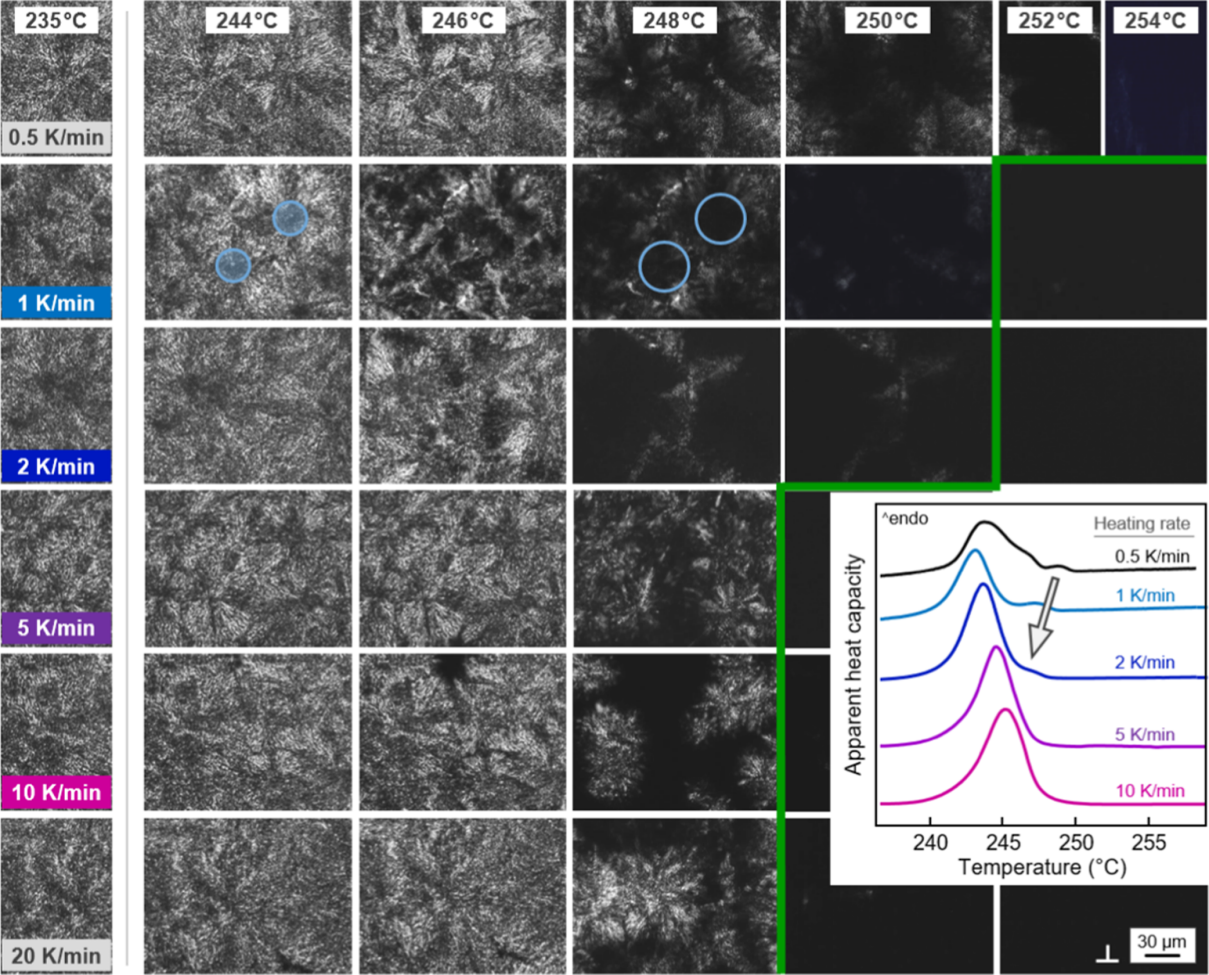
POM images captured during heating PBN containing β′-crystals isothermally formed at 235 °C (left column) at rates between 0.5 (top row) and 20 K/min (bottom row), with the polarizer directions indicated in the bottom right image. The inset, correspondingly, shows selected DSC heating scans, similarly recorded at rates between 0.5 (top) and 10 K/min (bottom). For each heating experiment, in both cases, POM and DSC analyses, a new sample was used. The scale bar holds for all images.

The melting behavior of PBN containing isothermally grown β′-crystals at different heating rates, as well as their stability, are further inspected with Figure 5, showing POM images of PBN spherulites formed at 235 °C, captured during heating at different rates between 0.5 (top row) and 20 K/min (bottom row), with the images to be read row-wise. The left column shows the POM structure of PBN after crystallization at 235 °C. These images reveal the presence of spherulites with their typical appearance if containing β′-phase, that is, absence of both a clear Maltese cross and well-defined birefringence along the radius direction, for reasons described above (see Figure 2). For an improved discussion of the POM images, the inset in Figure 5 shows DSC scans of PBN crystallized at 235 °C, recorded during heating at different rates, as indicated. The data reveal a large melting peak close to 245 °C and then—depending on the heating rate—further (up to three) weak melting events in the temperature range from 245 to 250 °C, partially only visible as a high-temperature shoulder on the main melting peak. The crystals which melt at high temperature fade with increasing heating rate and the corresponding melting peaks shift to lower temperature until merging with the main melting peak at around 245 °C on heating at 10 K/min, or disappear (see gray arrow). This observation indicates that the small high-temperature melting peaks are caused by reorganization of crystals originally formed at 235 °C.^18^

For rather high heating rates of 5, 10, and 20 K/min (see the bottom three rows), the images in Figure 5 confirm the observation described in Figure 3. In these cases, melting of β′-crystals formed at 235 °C begins at the edge of the spherulites, stretching continuously over a rather narrow temperature range and completing below 250 °C (see the green line, which demarks the approximate end of melting). This observation is consistent with the detection of a single DSC melting peak (see the bottom curves in the inset). We detected qualitatively different melting behavior if samples were heated at rates lower than 5 K/min (top three image rows). In such cases, melting does not begin at the spherulite edges rather than in their centers or in volumes near the center, as is illustrated with the light-blue circles in the heating experiment using a rate of 1 K/min. This observation is consistent with the detection of a high-temperature melting event in the DSC analysis. Both DSC and POM confirm that the melting of such crystals, which reorganized during slow heating, occurs around 250 °C, that is, a few K higher than the melting of crystals in the spherulite centers, which are unable to reorganize even at the lowest rate of 0.5 K/min applied in this work.

**Figure 6:**
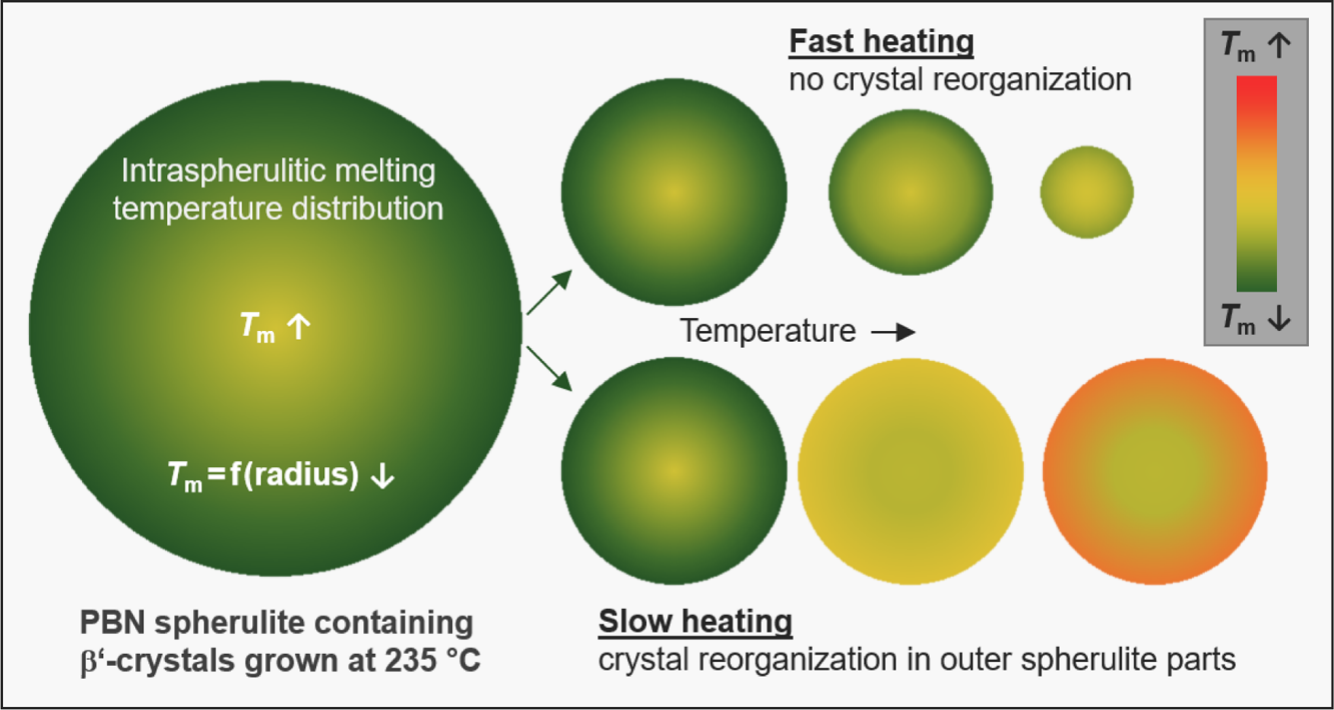
Sketch of melting of PBN spherulites containing β′-crystals at fast (top) and slow (bottom) heating. Colors/shading indicate local melting temperatures (see legend).

Figure 6 schematically summarizes the melting behaviors of PBN spherulites containing β′-crystals formed at 235 °C during fast (top row) and slow heating (bottom row). At a high heating rate, the spherulite gradually melts inward along the radius direction, indicating a higher stability of crystals in the spherulite center. Possible reasons may include a longer annealing time compared to crystals grown in the late stage of the crystallization process, allowing their stabilization, or a different morphology of crystals in the core compared to the spherulite outer regions. While for regions far from the center, a distinct superstructure composed of radially and tangentially grown lamellae is proven, such a structure may be absent in the core. The rather low stability of crystals in the outer regions of the spherulites translates into a more pronounced (compared to crystals in the spherulite core) stabilization/reorganization process, detectable on sufficiently slow heating (see bottom row sketches).

### Effect of Annealing/Secondary Crystallization on the Spatial Melting-Temperature Distribution in Spherulites

**Figure 7:**
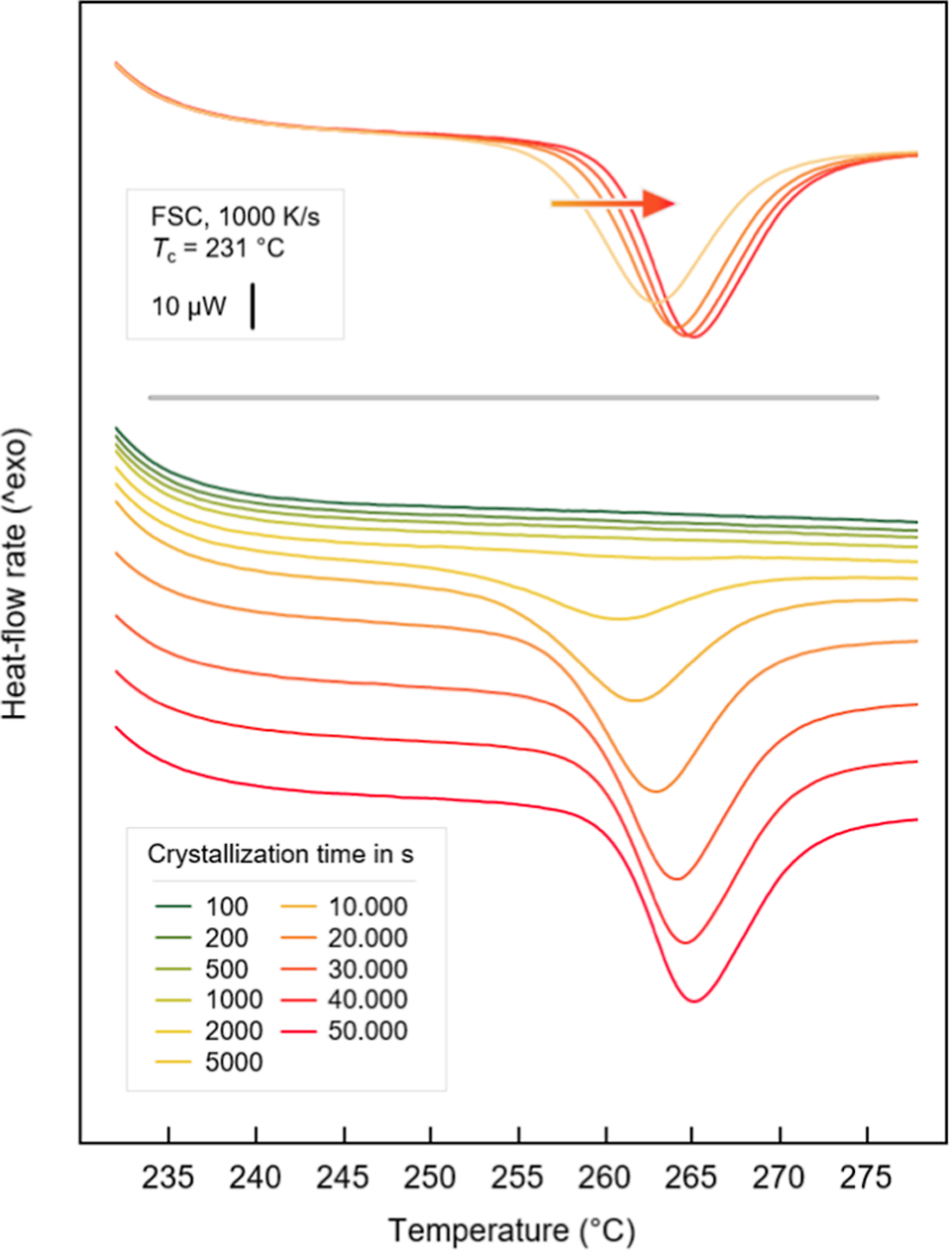
FSC heating curves of PBN crystallized for different times at 231 °C as indicated in the legend, recorded at 1000 K/s. For clarity, the lower panel shows the curves vertically shifted to each other. Selected curves, corresponding to crystallization times from 20,000 to 50,000 s, are shown superimposed in the upper panel, illustrating a shift of the entire melting peak during secondary crystallization.

We suggested above that the observed intraspherulitic melting-temperature distribution, causing inward melting of spherulites on fast heating, may be related to different isothermal annealing times of the center and outer regions of the spherulites during the course of the crystallization process, before the analysis of the melting temperature. While crystals located in the center of the spherulites were annealed for periods of time on the order of magnitude of minutes to several hours, crystals grown at the periphery of the spherulites were not annealed, presumably being therefore of lower perfection. In order to prove/disprove crystal perfection during isothermal crystallization, Figure 7 shows FSC heating curves of PBN, recorded at 1000 K/s, after crystallization at 231 °C for different times up to 50,000 s (around 13.9 h).

The curves reveal melting of crystals formed at 231 °C, with both the area and temperature of the melting peak increasing with the crystallization time (see the lower panel). Importantly, the increase of the melting temperature is not just visible as an increase of the temperature of the peak maximum, which besides structure effects is also controlled by instrumental factors (in this case the mass of crystals),^42^ but shows up as a shift of the entire melting event, illustrated in the upper panel of Figure 7 for curves recorded in the crystallization-time range of secondary crystallization and, presumably, after a space-filled spherulitic structure is achieved. Note that as long as the spherulites grow (primary crystallization), unstabilized crystals exist and contribute to a constant peak onset, with these curves not shown/considered here.

**Figure 8:**
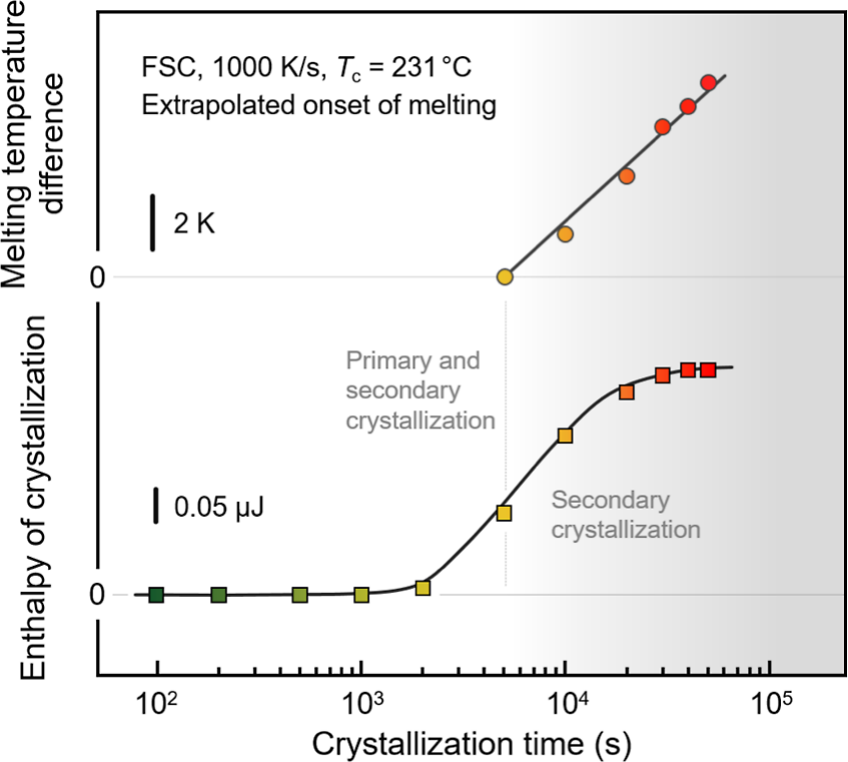
Enthalpy of crystallization (lower part) and relative increase of the melting temperature (extrapolated onset) (upper part) of PBN as a function of the time of crystallization at 231 °C, as deduced from the data of Figure 7. Color-coding of data points is in accord with colors of the FSC curves in Figure 7, and the gray-shaded part of the plots indicates the crystallization-time range of secondary crystallization only.

The enthalpy of crystallization equals the enthalpy of melting calculated from the area of the melting peaks in the FSC heating curves and is shown as a function of the crystallization time in the lower part of Figure 8, while the upper part shows the increase of the extrapolated onset of the melting temperature with crystallization time in the late stage of the crystallization process after space-filling.

Typically, the increase of the enthalpy of crystallization with crystallization time reveals the stages of primary and secondary crystallization, recognized by their different kinetics.^43−45^ For the high-temperature crystallization process of PBN investigated here, primary crystal growth strongly overlaps with secondary crystallization, with the increase of the onset melting temperature clearly indicating the completion of primary crystallization, as illustrated with the gray-shaded area in Figure 8.

In general, relatively fast primary/bulk crystallization is associated with growth of crystals in the bulk melt, while relatively slow secondary crystallization may involve perfection of crystals grown in the primary crystallization stage (mainly by so-called lamellar thickening)^46−48^ and/or the formation of additional crystals in amorphous regions between the primary crystals (often called insertion-crystallization).^49,50^ Insertion crystallization, occurring in interlamellar spaces, even on isothermal crystallization, is expected to produce crystals of lower stability/melting temperature compared to primary crystals,^48^ which, however, is not observed in the FSC heating scans of Figure 7. A special case of secondary crystallization is growth of daughter lamellae at the fold-surface of existing mother lamellae, being also possible in case of PBN in the shed of light of the morphological observations of Figures 2 and 3.^51^

As such, the data of Figures 7 and 8, in particular, the increase of the melting temperature with crystallization time, which in DSC analysis is a spatially over the entire sample volume averaged value, confirms the occurrence of secondary crystallization in PBN at the selected crystallization condition. At the present stage of research, we suggest that this process involves a time-controlled stabilization of all crystals within the spherulitic superstructure, regardless their radial or tangential orientation, with the latter assumption based on the observation of a single monomodal melting-temperature distribution only.

Regarding the specific data of Figures 7 and 8, we need acknowledging of the limited reproducibility of the experiments related to the small size of samples in FSC analyses, affecting the overall crystallization kinetics due to nonreproducible crystal nucleation. This causes uncertainties with respect to the reported crystallization times (legend in Figure 7, crystallization-time axis in Figure 8), however, with the conclusions derived from the data not affected. To gain ultimate confidence, numerous crystallization experiments in a rather wide range of crystallization temperatures were performed, confirming distinct isothermal secondary crystallization and the shift of the onset temperature of melting.

**Figure 9:**
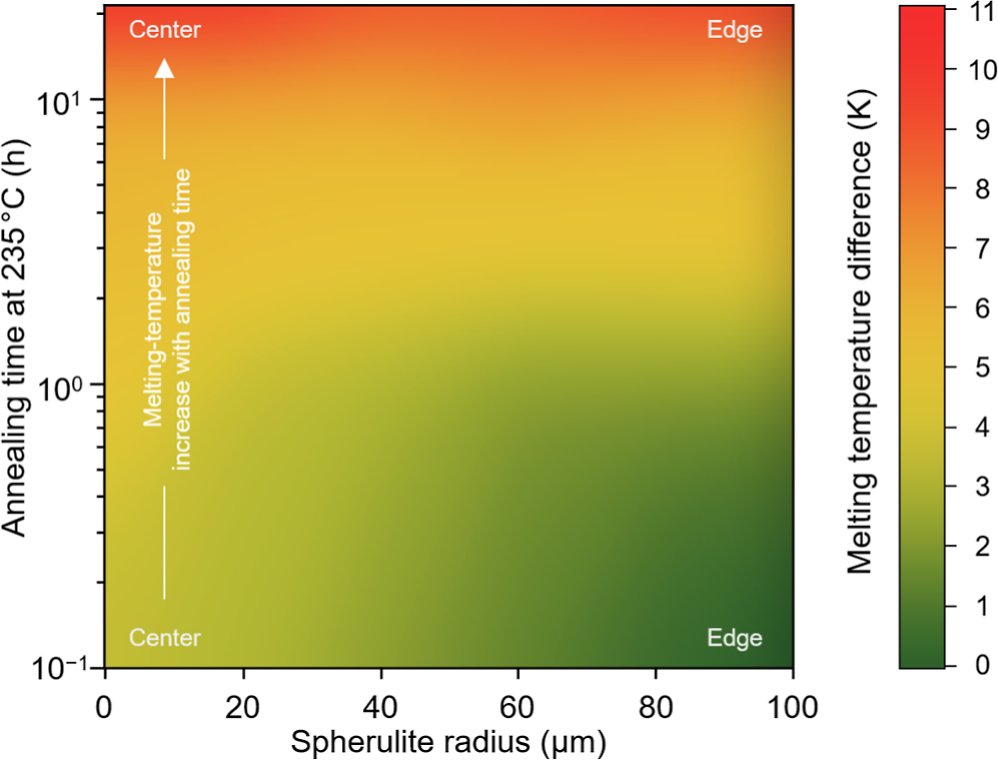
Color map of the relative melting temperature of PBN crystallized at 235 °C, containing β′-crystals, as a function of the spherulite radius (from center to edge, increasing from left to right) and of the time of annealing spherulites after space filling (increasing from bottom to top). Data are based on analysis of the local melting temperature within spherulites with a diameter of 200 μm, obtained on heating at 20 K/min.

This knowledge supports the initial idea of explaining inward melting of non-long-term aged PBN spherulites, containing β′-crystals, by a radial melting-temperature distribution caused by short-term aging of the inner spherulite parts, with the aging time inversely scaling with the spherulite radius. During the period of primary spherulite growth into the bulk melt, already formed crystals stabilize and exhibit a higher melting temperature than nonaged crystals at the spherulite boundary. Summarizing this observation, Figure 9 presents the local melting-temperature distribution within 200 μm-spherulites of PBN containing β′-crystals within a color map, as a function of the annealing time after a space-filled structure was achieved. The plot illustrates that immediately after space-filling (corresponding zero annealing time; see data in the lower part of the plot) the melting temperature in the spherulite center (left part) is 3–4 K higher than at the boundary (right part). With increasing annealing time (vertical axis), the melting-temperature gradient diminishes, such that the melting temperature is rather independent of the radius.

## Conclusions

This study provides information about the importance of secondary crystallization/annealing on the thermodynamic stability/melting behavior of crystals when arranged in a spherulitic semicrystalline superstructure, on example of β′-crystals of PBN formed around 235 °C. In this specific case, the melting temperature was assessed by heating at 20 K/min, suppressing crystal reorganization, which otherwise complicates the analysis of the melting characteristics. Slow growth of spherulites allows annealing and perfection/stabilization of crystals in the inner spherulite parts which, therefore, melt at a higher temperature than non-annealed crystals evident at the spherulite boundary. In the specific case of PBN β′-crystals, arranged in 200 μm sized spherulites, the melting-temperature difference may be a few K. With increasing time of secondary crystallization at the temperature of primary crystallization, the initial melting-temperature gradient along the spherulite radius diminishes and a single but increased melting temperature is evident. Though long-term annealing at the crystallization temperature yields a position-independent intraspherulitic melting temperature, cooling the system after completed primary isothermal crystallization, or nonisothermal spherulite growth may preserve/freeze such intraspherulitic melting temperature distribution with implications on the further properties. Worth noting, Fourier-transform infrared microscopy allowed detection of an intraspherulitic crystallinity gradient in case of PLLA when spherulites are subjected to a similar growth history (reorganized and nonreorganized spherulite core and edge, respectively) as applied here for PBN.^52^ In that case even a link to a gradient/radius dependence of nanoscale mechanical properties was proven, which suggests a wider impact of the observations presented for PBN in the present work.^52^

In addition to the analysis of the interplay between secondary crystallization and intraspherulitic melting, growth of β′-crystal lamellae at 235 °C with their long direction parallel and tangential to the spherulite radius is detected, with the radially oriented lamellae advancing the formation of tangential lamellae and suggesting that the growth of the latter may be nucleated at the surface of radial lamellae.
